# Quality control validation for a veterinary laboratory network of six Sysmex XT‐2000*i*V hematology analyzers

**DOI:** 10.1111/vcp.13163

**Published:** 2022-08-03

**Authors:** Susan Daly, Peter A. Graham, Kathleen P. Freeman

**Affiliations:** ^1^ SYNLAB‐VPG/Cork, South Cork Industrial Estate Cork Ireland; ^2^ School of Veterinary Medicine and Science University of Nottingham Leicestershire UK; ^3^ SYNLAB‐VPG/Exeter, Unit 1b, Science Park Exeter UK

**Keywords:** harmonization, hematology, quality control, sigma metrics, validation, Westgard rules

## Abstract

**Background:**

Quality control (QC) validation is an important step in the laboratory harmonization process. This includes the application of statistical QC requirements, procedures, and control rules to identify and maintain ongoing stable analytical performance. This provides confidence in the production of patient results that are suitable for clinical interpretation across a network of veterinary laboratories.

**Objectives:**

To determine that a higher probability of error detection (*P*_ed_) and lower probability of false rejection (*P*_fr_) using a simple control rule and one level of quality control material (QCM) could be achieved using observed analytical performance than by using the manufacturer's acceptable ranges for QCM on the Sysmex XT‐2000*i*V hematology analyzers for veterinary use. We also determined whether Westgard Sigma Rules could be sufficient to monitor and maintain a sufficiently high level of analytical performance to support harmonization.

**Methods:**

EZRules3 was used to investigate candidate QC rules and determine the *P*_ed_ and *P*_fr_ of manufacturer's acceptable limits and also analyzer‐specific observed analytical performance for each of the six Sysmex analyzers within our laboratory system using the American Society of Veterinary Clinical Pathology (ASVCP)‐recommended or internal expert opinion quality goals (expressed as total allowable error, TE_a_) as the quality requirement. The internal expert quality goals were generated by consensus of the Quality, Education, Planning, and Implementation (QEPI) group comprised of five clinical pathologists and seven laboratory technicians and managers. Sigma metrics, which are a useful monitoring tool and can be used in conjunction with Westgard Sigma Rules, were also calculated.

**Results:**

The QC validation using the manufacturer's acceptable limits for analyzer 1 showed only 3/10 measurands reached acceptable *P*_ed_ for veterinary laboratories (>0.85). For QC validation based on observed analyzer performance, the *P*_ed_ was >0.94 using a 1‐2.5s QC rule for the majority of observations (57/60) across the group of analyzers at the recommended TE_a_. We found little variation in *P*_fr_ between manufacturer acceptable limits and individual analyzer observed performance as this is a characteristic of the rule used, not the analyzer performance.

**Conclusions:**

An improved probability of error detection and probability of false rejection using a 1‐2.5s QC rule for individual analyzer QC was achieved compared with the use of the manufacturers' acceptable limits for hematology in veterinary laboratories. A validated QC rule (1‐2.5s) in conjunction with sigma metrics (>5.5), desirable bias, and desirable CV based on biologic variation was successful to evaluate stable analytical performance supporting continued harmonization across the network of analyzers.

## 1 INTRODUCTION

Quality goals have been established for clinical laboratory procedures based on biological variation data or expert opinion,^1^, ^2^, ^3^, ^4^, ^5^ and analytical equipment that achieves these quality goals will generate results that are suitable for clinical decision‐making.^6^ Once analyzers have been evaluated and optimized to meet quality goals, statistical quality control (QC) aims to maintain performance within those goals. Statistical QC relies on one or more control rule applied to the results generated from regular analyses of quality control materials (QCM). For statistical QC to be effective in achieving its purpose, the selected rule(s) must be sensitive and specific for the identification of deteriorating analytical performance. That is, statistical control validation is a necessary step in the design and implementation of statistical QC rule(s).

In a network of veterinary laboratory hematology analyzers that have undergone a harmonization process,^7^ statistical QC performs an additional role in maintaining harmonization and allowing for continued interchangeability of results and common reference intervals within the veterinary network. In the same way that QC validation supports the effectiveness of QC to ensure the stability of a single analyzer, QC validation is a necessary step in the design and implementation of QC rules for a network of harmonized analyzers.

The sensitivity and specificity of QC are reflected by the probability of error detection (*P*_ed_) and probability of false rejection (*P*_fr_), respectively. The *P*_ed_ is a measure of the frequency with which a control rule would cause analytical runs to be rejected when results contain errors beyond the inherent imprecision. Ideally, error detection should be set to 100% for medically significant errors; however, error detection at ≥90% is considered sufficient.^6^ Conversely, *P*_fr_ is a measure of the frequency with which analytical runs are rejected when there is no apparent reason or issue. The goal is to have the highest possible *P*_ed_ to ensure that medically important errors are not missed and a low *P*_fr_ (≤5%)^6^ to reduce the waste cost and efficiency impact on patient sample volume, reagent use, and result turnaround time. This optimizes the efficiency and capability of statistical QC as a tool for the demonstration of stable laboratory system performance. The analytical performance capabilities of the analyzer inform the choice of the number of control materials and statistical rules selected, which then determines the *P*_ed_ and *P*_fr_ achieved. This step‐by‐step process results in a quality control validation.

Laboratories using statistical QC often employ the Westgard rules, which are denoted by a shorthand notation. For example, a 1‐2s rule means that control limits encompass two standard deviations (SD) on each side of the observed mean. A 1‐3s refers to a rule that is set at ±3 SDs, and the rule is violated when one measurement exceeds ±3 SDs from the QCM mean. The numeral 2 placed in front of a rule, such as 2‐2s, indicates the rule is violated when two consecutive control measurements or two single measurements across two QCMs are outside the 2SD control limits.^6^

Six Sigma process‐improvement methodology has been applied to clinical laboratory analyses such that performance capability (bias and imprecision) relative to TE_a_ can be represented on a numeric scale as a “sigma metric” (σ). Sigma metrics and QC rules have been combined in Westgards Sigma Rules,^6^ such that sigma metrics can, in turn, determine whether a simple single QC rule or a more complicated collection of rules is required. A sigma metric ≥6 for an analytical method indicates <3.4 errors/defects per million results,^8^ defined as world‐class performance, and the implied low bias and imprecision mean that these methods are easily controlled with simple QC rules and a low number of QCM measurements. It would take a large deterioration in performance to cause a clinical error, and such a large shift could be readily detected with a simple QC rule. In fact, for measurands with sigma metrics ≥6.0, acceptable *P*_ed_ and *P*_fr_ can be achieved using 1 or 2 control measurements. Conversely, measurands with lower sigma metrics require a multirule and/or larger number of control measurements and may or may not be able to achieve acceptable *P*_ed_ and *P*_fr_.^9^ Commercially available software called EZRules3 (Westgard QC, https://www.westgard.com/store/software.html) allows the user to explore and select QC rules based on the total error quality goal and observed analytical performance as well as the *P*_ed_ and *P*_fr_ that can be achieved.^8^

It has been acknowledged that a *P*_ed_ of ≥0.85 can be used^10^ for point of care testing (POCT) in veterinary medicine or for hematologic analyses, where the use of a single QCM is preferred. The use of a single hematology control material is traditional in the UK but a *P*_ed_ ≥0.90 cannot be achieved with a 1‐3s rule and a single QCM data point can only achieve a *P*_ed_ ≥0.85.^11^, ^12^

This paper describes a network of six harmonized Sysmex XT‐2000*i*V hematology analyzers across five locations,^7^ which required a QC approach that could ensure the maintenance of individual analyzer performance and supported continued harmonization. In deciding which QC approaches to evaluate, the following preferences and constraints had to be taken into consideration. First, the use of a simple control rule (e.g., 1‐2.5s, 1‐3s) rather than a multirule and a single level of QCM was preferred due to the simplicity of training and evaluation and based on traditional laboratory practices and economics. Second, the choice of QCM was limited because a third‐party control material was not available for the Sysmex analyzer, only a manufacturer‐provided QCM. It was felt that the potential disadvantages of a single level of QCM could be mitigated since all blood smears undergo additional nonstatistical quality control via microscopy by a fully trained technician to validate the automated results prior to a pathologist's review of laboratory data and correlation with clinical findings before results are reported.

The authors set out to discover whether an effective QC approach supporting network harmonization could be successfully implemented, given these constraints and preferences. This study addressed the following objectives:

1. Determine that a higher *P*_ed_ can be achieved using QC rules validated for each individual analyzer than that achieved using the manufacturer's acceptable limits for the control limits.
2. Determine that a *P*_ed_ (≥0.85) and *P*_fr_ (≤0.05) can be achieved using a single QCM (*n* = 1) with a simple single control rule (1‐2.5s or 1‐3s).
3. Assess the use of sigma metric evaluation as part of the QC approach in conjunction with the validated QC rules appropriate for each analyzer, with a goal of using it to monitor when instrument servicing is needed to maintain a high level of instrument performance.
4. Assess whether analyzer performance criteria established in a previous study (bias <3%, achievement of desirable biologic variation‐based goals for CV and bias, and 0.33CV_I_ goals, with sigma metrics >5) would be confirmed in this study as useful contributions to an overall QC approach.

## 2 MATERIAL AND METHODS

### 2.1 Hematology analyzers

The data from six Sysmex XT‐2000*i*V analyzers (Sysmex Corporation, Kobe, Japan) located in five laboratory locations in the UK (*n* = 4) and Ireland (*n* = 1) were evaluated. Analyzers were designated analyzers 1, 2, 3, 4a, 4b, and 5. This network of analyzers had previously undergone optimization and successful harmonization.^7^ Analyzer 1 was designated the reference analyzer.

### 2.2 Quality control material

The QCM used for these evaluations was Level 2—Normal e‐CHECK (XE)‐Hematology Control (Sysmex Corporation) provided in an 8‐vial kit with one new vial used per week. Typically the lot number changed for each new kit with slight changes in the manufacturer's target means and acceptable limits. A single lot number was used for all analyzers during the study. The hematologic measurands evaluated were the white blood cell count (WBC), red blood cell count (RBC), hemoglobin concentration (HGB), hematocrit (HCT), mean cell volume (MCV), mean cell hemoglobin concentration (MCHC), reticulocyte count (RETIC), platelet count (PLT), plateletcrit (PCT), and red cell distribution width‐coefficient of variation (RDW‐CV). The QCM was analyzed by a fully trained technician according to standard operating procedures; when not in use, the QCM vial was refrigerated.

### 2.3 Evaluation of manufacturer's acceptable limits as control rules using EZRules3

The manufacturer's acceptable limits for the hematologic measurands were evaluated using data from 1 month of QCM results from analyzer 1. The width of the manufacturer's acceptable limits for a measurand was divided by the standard deviation (SD) for the reference lab (analyzer 1) to determine the number of SDs contained within this range. That number was divided by two to determine the SDs on each side of the target mean and was then rounded to the nearest QC rule available within EZRules3 to evaluate the performance of the control rule most closely representative of the manufacturer's acceptable limit. EZRules3 allows the manual selection of simple control rules for 1‐2s, 1‐2.5s, 1‐3s, 1‐3.5s, 1‐4s, 1‐5s, and 1‐6s. If the manufacturer's range control rule was equivalent to <±2SDs, a 1‐2s rule was used. If the manufacturer's range control rule was >6 SDs, a 1‐6s rule was used.

The control rule was then assessed using EZRules3 to determine the *P*_ed_ and *P*_fr_ that are possible using the startup QC design, which is a program that allows the user to follow a series of prompts, including the manufacturer's target mean as the decision‐level concentration, ASVCP recommendations,^3^ and/or internal expert opinion for total allowable error (TE_a_) as the chosen quality requirement, and the number of QCMs set to *n* = 1. For those measurands, where no ASVCP recommendation for TE_a_ was available (PCT and RETIC number), expert opinion goals from an internal working group composed of pathologists and technicians were used (5 and 7 persons, respectively). The expected instability setting in the software was set to off.

### 2.4 QC validation—evaluation of control rules based on observed individual analyzer performance

The QC validation for each of the six Sysmex instruments, customized for observed performance, was determined using approximately 1 month of QC data (March 2020), as reported previously.^7^ We used EZRules3 to plot the observed imprecision and observed bias (calculated from the manufacturer's target mean) generated from daily repeated QC measurements for each analyzer and measurand to determine individual analyzer QC rules and associated *P*_ed_, *P*_fr_, and sigma metrics.

The candidate rules evaluated using the manual selection option were 1‐2.5s, *n* = 1 and 1‐3s, *n* = 1. A *P*_ed_ ≥0.90 can theoretically be achieved with a 1‐2.5s rule and a single QCM if performance is sufficiently good.

### 2.5 Review of criteria for measurands with poor performance

Findings from a previous study^7^ indicated that Sigma metrics >5, bias <3%, and measurands achieving desirable biologic variation goals for CV and bias demonstrated confident stable analytical performance. These criteria were reviewed and compared with the findings of this study, which confirmed that harmonization was maintained during the course of the study.

## 3 RESULTS

### 3.1 QC validation for manufacturer's acceptable limits

The results of the QC validation for the manufacturer's acceptable limits for the QCM using the observed SD for analyzer 1 are summarized in Table 1. Acceptable criteria for *P*_ed_ (>0.85) were met for only three measurands (HGB, PCT, and WBC). Acceptable *P*_fr_ (≤0.05) was achieved for all 10 measurands using the available control rules that most closely represented the manufacturer's acceptable limits.

**TABLE 1:** Summary of QC validation for Sysmex quality control Level 2, Manufacturer's acceptable QCM ranges using 1 month of QC data from the network reference laboratory Sysmex XT 2000*i*V hematology analyzer (analyzer 1)

Measurand (units)	RBC (×10^6^/μL)	HBG (g/dl)	HCT (%)	MCV (fL)	MCHC (g/dL)	RDW‐CV (%)	RETIC (×10^6^/μL)	WBC (×103/μL)	PLT (×103/μL)	PCT (%)
Manufacturer's acceptable lower limit	4.05	11.3	32.7	76.7	31.7	13.8	63.8	6.81	188	15
Manufacturer's acceptable upper limit	4.47	11.9	36.1	84.7	35.7	18.6	132.6	7.69	254	27
Width of acceptable range	0.42	0.6	3.4	8.0	4.0	4.8	68.8	0.88	66	12
Observed SD for analyzer 1	0.03	0.15	0.35	0.44	0.32	0.09	3.71	0.16	6.50	0.79
Number of observed SD within the acceptable range	14.00	4.00	9.70	18.18	12.50	53.33	18.54	5.50	10.15	1.27
Number of observed SD on each side of the target mean	7.00	2.00	4.85	9.09	6.25	26.66	9.27	2.75	5.07	0.63
Closest available control rule	1‐6s	1‐2s	1‐5s	1‐6s	1‐6s	1‐6s	1‐6s	1‐3s	1‐5s	1‐2s
TE_a_% (ASVCP except for PCT and RETIC #)	10	10	10	7	10	10	40	15	20	25
Observed CV % (analyzer 1)	0.66	1.32	1.00	0.54	0.96	0.54	5.81	2.12	2.87	3.65
Observed bias % (analyzer 1)	0.008	0.44	0.66	0.75	1.05	0.19	4.00	1.16	2.33	2.89
*P*_ed_	>0.01	>0.98	>0.13	>0.01	>0.01	>0.01	>0.01	>0.85	>0.13	>0.98
*P*_fr_	0.0	0.05	0	0	0	0	0	0	0	0.05
Sigma metric	15.14	7.24	9.34	11.57	9.32	18.17	6.20	6.53	6.16	6.06

*Notes*: Number of observed SDs in the manufacturer's acceptable range was divided by two to determine the number of SD on each side of the manufacturer's target mean. This was used to determine the closest control rule available for selection in the EZRULES3 program for evaluation. If the number of SD within the acceptable range/2 is much less than the closest available control rule, the width of the acceptable range will be reduced compared with the manufacturer's acceptable range, resulting in overestimation of the *P*_ed_ (PCT). If the SD within the acceptable range/2 is much greater than the closest available control rule, the width of the acceptable range will be greater than the manufacturer's acceptable range, resulting in underestimation of the *P*_ed_ (RDW‐CV).

Abbreviations: ASVCP, the American Society of Veterinary Clinical Pathology; CV, coefficient of variation; HBG, hemoglobin; HCT, hematocrit; MCHC, Mean Corpuscular Hemoglobin Concentration; MCV, Mean Corpuscular Volume; *P*_ed_, probability of error detection; *P*_fr_, probability of false rejection; PLT, Platelet count; PCT, Plateletcrit QC, quality control; QCM, quality control material; RBC, Red Blood Cell count; RDW‐CV, coefficient of variation of the Red Cell Distribution Width; RETIC, reticulocyte count; SD, standard deviation; TE_a_, total allowable error; WBC = White Blood Cell count.

### 3.2 QC validation based on observed analyzer performance for six Sysmex analyzers

Tables 2, 3, 4 summarize the results of the QC validation for two control rules (1‐2.5s and 1‐3s) based on observed analyzer performance (March 2020) for the selected measurands on six Sysmex analyzers. Only three measurands did not achieve a *P*_ed_ ≥0.85; RBC and PLT on analyzer 4a using a 1‐3s rule (*n* = 1), and PCT using both QC rules, 1‐2.5s and 1‐3s (*n* = 1). All other measurands achieved a *P*_ed_ ≥0.85 with either control rule across the six analyzers.

**TABLE 2:** Summary of the hematological erythroid measurand QC validation for six Sysmex XT 2000*i*V hematology analyzers based on observed performance (March 2020)

Hematology analyzers
Analyzer 1		Analyzer 2		Analyzer 3		Analyzer 4a		Analyzer 4b		Analyzer 5	
RBC (×10^6^/μL)												
TE_a_ goal %	10		10		10		10		10		10	
Observed mean	4.32		4.37		4.34		4.39		4.39		4.35	
Target mean	4.32		4.32		4.32		4.32		4.32		4.32	
Observed CV %	0.69		0.49		0.68		1.60		0.70		1.00	
Observed absolute bias %	0.10		1.13		0.40		1.60		1.58		0.64	
Observed TE	1.48		2.11		1.76		4.8		2.98		2.64	
Sigma metric	14.35		18.1		14.12		5.25		12.03		9.36	
Control rules, *n* = 1	1‐2.5s	1‐3s	1‐2.5s	1‐3s	1‐2.5s	1‐3s	1‐2.5s	1‐3s	1‐2.5s	1‐3s	1‐2.5s	1‐3s
*P*_ed_	>0.94	>0.85	>0.94	>0.85	>0.94	>0.85	0.88	**0.76**	>0.94	>0.85	>0.94	>0.85
*P*_fr_	0.01	0.00	0.01	0.00	0.01	0.00	0.01	0.00	0.01	0.00	0.01	0.00
HGB (g/dL)												
TE_a_ goal %	10		10		10		10		10		10	
Observed mean	12.08		11.90		11.98		12.21		12.22		12.11	
Target mean	12		12		12		12		12		12	
Observed CV %	0.59		0.37		0.32		0.47		0.44		0.90	
Observed absolute bias %	0.63		0.80		0.15		1.79		1.87		0.90	
Observed TE	1.81		1.54		0.79		2.73		2.75		2.7	
Sigma metric	15.88		24.86		30.78		17.47		18.48		10.11	
Control rules, *n* = 1	1‐2.5s	1‐3s	1‐2.5s	1‐3s	1‐2.5s	1‐3s	1‐2.5s	1‐3s	1‐2.5s	1‐3s	1‐2.5s	1‐3s
*P*_ed_	>0.94	>0.85	>0.94	>0.85	>0.94	>0.85	>0.94	>0.85	>0.94	>0.85	>0.94	>0.85
*P*_fr_	0.01	0.00	0.01	0.00	0.01	0.00	0.01	0.00	0.01	0.00	0.01	0.00
TE_a_ goal %	10		10		10		10		10		10	
HCT (%)												
TE_a_ goal %	10		10		10		10		10		10	
Observed mean	36.1		36.5		35.9		36.3		36.6		36.3	
Target mean	35.7		35.7		35.7		35.7		35.7		35.7	
Observed CV %	0.64		0.56		1.06		0.55		0.47		1.01	
Observed absolute bias %	1.14		2.13		0.43		1.78		2.40		2.05	
Observed TE	2.42		3.25		2.55		2.88		3.34		4.07	
Sigma metric	13.84		14.11		9.03		14.95		16.17		7.87	
Control rules, *n* = 1	1‐2.5s	1‐3s	1‐2.5s	1‐3s	1‐2.5s	1‐3s	1‐2.5s	1‐3s	1‐2.5s	1‐3s	1‐2.5s	1‐3s
*P*_ed_	>0.94	>0.85	>0.94	>0.85	>0.94	>0.85	>0.94	>0.85	>0.94	>0.85	>0.94	>0.85
*P*_fr_	0.01	0.00	0.01	0.00	0.01	0.00	0.01	0.00	0.01	0.00	0.01	0.00
MCV (fL)												
TE_a_ goal %	7		7		7		7		7		7	
Observed mean	83.5		83.4		82.7		83.7		83.4		83.8	
Target mean	82.6		82.6		82.6		82.6		82.6		82.6	
Observed CV %	0.43		0.40		0.69		0.55		0.57		0.62	
Observed absolute bias %	1.09		1.04		0.08		0.92		0.92		1.45	
Observed TE	1.95		1.84		1.46		2.02		2.06		2.69	
Sigma metric	13.74		14.90		10.03		11.05		11.72		8.95	
Control rules, *n* = 1	1‐2.5s	1‐3s	1‐2.5s	1‐3s	1‐2.5s	1‐3s	1‐2.5s	1‐3s	1‐2.5s	1‐3s	1‐2.5s	1‐3s
*P*_ed_	>0.94	>0.85	>0.94	>0.85	>0.94	>0.85	>0.94	>0.85	>0.94	>0.85	>0.94	>0.85
*P*_fr_	0.01	0.00	0.01	0.00	0.01	0.00	0.01	0.00	0.01	0.00	0.01	0.00
MCHC (g/dL)												
TE_a_ goal %	10		10		10		10		10		10	
Observed mean	33.4		32.6		33.4		33.5		33.3		33.3	
Target mean	33.6		33.6		33.6		33.6		33.6		33.6	
Observed CV %	0.70		0.72		1.11		0.92		0.74		0.83	
Observed absolute bias %	0.50		0.54		0.54		0.27		0.68		1.08	
Observed TE	1.9		1.98		2.76		2.11		2.16		2.74	
Sigma metric	13.57		13.14		8.52		10.58		12.59		11.89	
Control rules, *n* = 1	1‐2.5s	1‐3s	1‐2.5s	1‐3s	1‐2.5s	1‐3s	1‐2.5s	1‐3s	1‐2.5s	1‐3s	1‐2.5s	1‐3s
*P*_ed_	>0.94	>0.85	>0.94	>0.85	>0.94	>0.85	>0.94	>0.85	>0.94	>0.85	>0.94	>0.85
*P*_fr_	0.01	0.00	0.01	0.00	0.01	0.00	0.01	0.00	0.01	0.00	0.01	0.00
RDW‐CV (%)												
TE_a_ goal %	10		10		10		10		10		10	
Observed mean	16.1		16.2		16.1		16.2		16.3		16.3	
Target mean	16.2		16.2		16.2		16.2		16.2		16.2	
Observed CV %	0.48		0.75		0.49		0.69		0.56		0.64	
Observed absolute bias %	0.69		0.09		0.33		0.02		0.59		0.57	
Observed TE	1.65		1.59		1.31		1.4		1.71		1.85	
Sigma metric	19.40		13.21		19.73		14.46		16.80		14.73	
Control rules, *n* = 1	1‐2.5s	1‐3s	1‐2.5s	1‐3s	1‐2.5s	1‐3s	1‐2.5s	1‐3s	1‐2.5s	1‐3s	1‐2.5s	1‐3s
*P*_ed_	>0.94	>0.85	>0.94	>0.85	>0.94	>0.85	>0.94	>0.85	>0.94	>0.85	>0.94	>0.85
*P*_fr_	0.01	0.00	0.01	0.00	0.01	0.00	0.01	0.00	0.01	0.00	0.01	0.00
RETIC (×10^6^/μL)												
TE_a_ goal %	40		40		40		40		40		40	
Observed mean	70.1		73.9		71.8		76.5		77.0		75.9	
Target mean	74.3		74.3		74.3		74.3		74.3		74.3	
Observed CV %	5.11		4.58		4.30		4.87		6.07		4.73	
Observed absolute bias %	4.65		3.37		3.37		2.91		3.72		0.44	
Observed TE	14.87		12.53		11.97		12.65		15.86		9.9	
Sigma metric	6.48		8		8.52		7.62		5.98		8.36	
Control rules, *n* = 1	1‐2.5s	1‐3s	1‐2.5s	1‐3s	1‐2.5s	1‐3s	1‐2.5s	1‐3s	1‐2.5s	1‐3s	1‐2.5s	1‐3s
*P*_ed_	>0.94	>0.85	>0.94	>0.85	>0.94	>0.85	>0.94	>0.85	>0.94	>0.85	>0.94	>0.85
*P*_fr_	0.01	0.00	0.01	0.00	0.01	0.00	0.01	0.00	0.01	0.00	0.01	0.00

*Notes*: All instruments used the same QC material lot number. The number of QC materials is one (*n* = 1). *P*_ed_ <0.85 is highlighted in bold type.

Abbreviations: CV, coefficient of variation; HBG, hemoglobin; HCT, hematocrit; MCHC, mean corpuscular hemoglobin concentration; MCV, mean corpuscular volume; *P*_ed_, probability of error detection; *P*_fr_, probability of false rejection; QC, quality control; RBC, red blood cell count; RDW‐CV, coefficient of variation of the red cell distribution width; RETIC, reticulocyte count; TE, total error; TE_a_, total allowable error.

**TABLE 3:** Summary of PLT and PCT QC Validation for six Sysmex XT 2000*i*V hematology analyzers based on observed performance (March 2020)

Hematology analyzers												
	Analyzer 1		Analyzer 2		Analyzer 3		Analyzer 4a		Analyzer 4b		Analyzer 5	
PLT (×10^3^/μL)												
TE_a_ goal %	20		20		20		20		20		20	
Observed mean	220.4		220.0		209.4		217.2		224.1		213.1	
Target mean	220		220		220		220		220		220	
Observed CV %	2.61		2.21		2.23		3.72		2.48		2.21	
Observed absolute bias %	0.16		0.00		4.81		0.93		1.87		3.13	
Observed TE	5.38		4.42		9.27		8.37		6.83		7.55	
Sigma metric	7.6		9.05		6.81		5.13		7.31		7.63	
Control rules, *n* = 1	1‐2.5s	1‐3s	1‐2.5s	1‐3s	1‐2.5s	1‐3s	1‐2.5s	1‐3s	1‐2.5s	1‐3s	1‐2.5s	1–3 s
P_ed_	>0.94	>0.85	>0.94	>0.85	>0.94	>0.85	0.86	**0.72**	>0.94	>0.85	>0.94	>0.85
P_fr_	0.01	0.00	0.01	0.00	0.01	0.00	0.01	0.00	0.01	0.00	0.01	0.00
PCT (%)												
TE_a_ goal %	25		25		25		25		25		25	
Observed mean	0.21		0.22		0.20		0.21		0.21		0.20	
Target mean	0.21		0.21		0.21		0.21		0.21		0.21	
Observed CV %	3.09		3.27		2.38		5.31		2.98		2.83	
Observed absolute bias %	2.02		0.91		6.16		0.76		1.45		2.20	
Observed TE	8.2		7.45		10.92		11.38		7.41		7.86	
Sigma metric	7.44		7.37		7.92		4.56		7.9		8.06	
Control rules, *n* = 1	1‐2.5s	1‐3s	1‐2.5s	1‐3s	1‐2.5s	1‐3s	1‐2.5s	1‐3s	1‐2.5s	1‐3s	1‐2.5s	1–3 s
P_ed_	>0.94	>0.85	>0.94	>0.85	>0.94	>0.85	**0.69**	**0.50**	>0.94	>0.85	>0.94	>0.85
P_fr_	0.01	0.00	0.01	0.00	0.01	0.00	0.01	0.00	0.01	0.00	0.01	0.00

*Notes*: All instruments used the same QC material lot number. The number of quality control materials is one (*n* = 1). P_ed_ <0.85 is highlighted by bold type.

Abbreviations: CV, coefficient of variation; *P*_ed_, probability of error detection; *P*_fr_, probability of false rejection; PCT, plateletcrit; PLT, platelet count; QC, quality control; TE, total error; TE_a_, total allowable error.

**TABLE 4:** Summary of WBC QC validation for six Sysmex XT 2000*i*V hematology analyzers based on observed performance (March 2020)

Hematology analyzers	Analyzer 1		Analyzer 2		Analyzer 3		Analyzer 4a		Analyzer 4b		Analyzer 5	
WBC (×10^3^/μL)												
TE_a_ goal %	15		15		15		15		15		1	
Observed mean	7.27		7.17		7.12		7.10		7.16		7.16	
Target mean	7.11		7.11		7.11		7.11		7.11		7.11	
Observed CV %	1.19		1.82		2.31		1.62		1.78		1.38	
Observed absolute bias %	2.33		0.82		0.18		0.04		0.77		0.70	
Observed TE	4.71		4.46		4.8		3.28		4.33		3.46	
Sigma metric	10.65		7.79		6.42		9.23		7.99		10.36	
Control rules, *n* = 1	1‐2.5s	1‐3s	1‐2.5s	1‐3s	1‐2.5s	1‐3s	1‐2.5s	1‐3s	1‐2.5s	1‐3s	1‐2.5s	1‐3s
*P*_ed_	>0.94	>0.85	>0.94	>0.85	>0.94	>0.85	>0.94	>0.85	>0.94	>0.85	>0.94	>0.85
*P*_fr_	0.01	0.00	0.01	0.00	0.01	0.00	0.01	0.00	0.01	0.00	0.01	0.00

*Notes*: All instruments used the same QC material lot number. The number of QC materials is one (*n* = 1).

Abbreviations: CV, coefficient of variation; *P*_ed_, probability of error detection; *P*_fr_, probability of false rejection; QC, quality control; TE, total error; TE_a_, Total allowable error; WBC, white blood cell count.

*P*_fr_ ≤0.05 was achieved for all 10 measurands for each of the six Sysmex analyzers and both candidate control rules.

Sigma metrics were >6 for 56/60 observations. When the sigma metrics was <5.5, three measurands did not achieve a *P*_ed_ >0.85 (RBC, PLT, and PCT, as above for analyzer 4a); RETIC (σ = 5.98) on analyzer 4b did achieve an acceptable *P*_ed_ (>0.85).

### 3.3 Evaluation of QC rules

The 1‐2.5s rule offered the highest *P*_ed_ for all measurands for the group of Sysmex analyzers based on the observed analyzer performance and yielded *P*_fr_s of only 1% (Tables 2, 3, 4). The manufacturer's QC limits achieved acceptable *P*_ed_ for only three measurands (Table 1).

### 3.4 Sigma metric monitoring

A monthly review of sigma metrics showed three measurands that performed <5.5 sigma failed to achieve quality goals for *P*_ed_ for one (RBC and PLT) or both QC rules (PCT). On further investigation, the quality goal index (QGI)^13^ demonstrated that observed imprecision was implicated for two measurands, while imprecision and bias were implicated for one measurand. The same analyzer (4a) accounted for all three measurands with poor performance, which was compared with the other analyzers, where performance was >5.5 sigma. (see Tables 2, 3, 4).

### 3.5 Review and optimization of criteria for measurands with poor performance

Previous criteria established for stable analytical performance indicated that measurands with a bias <3% achieved desirable biologic variation‐based goals for CV, bias, and 0.33CVi goals, with sigma metrics >5 supported analytical stability and harmonization. In this study, we noted that a sigma metric >5.5 rather than 5 demonstrated analytical stability. *P*_ed_ was achieved when the observed CV and Bias achieved desirable biologic variation goals, as previously reported.^7^

Imprecision limits to achieve acceptable *P*_ed_ (>0.85) for RBC (see Table 2, analyzer 4a) using the 1‐2.5s rule; if desirable bias based on biologic variation was applied (1.761%), then the observed CV % must be ≤1.45 to achieve a *P*_ed_ >0.94. Similarly, for PLT on analyzer 4a (Table 3), the Sigma metric was 5.13, and for the 1‐2.5s rule, we could determine that if desirable bias was achieved based on biologic variation (5.17%), then the observed CV % must be ≤2.60% (3.72%). Therefore, the 1‐3s rule was not satisfactory, the 1‐2.5s rule was satisfactory at 0.86 *P*_ed_, and the other analyzers achieved a *P*_ed_ >0.94.

## 4 DISCUSSION

To the authors' knowledge, this is the first reported QC validation for a network of Sysmex analyzers used in veterinary hematology and ongoing harmonization within a laboratory system. The quality goal used to determine the *P*_ed_ and *P*_fr_ was TE_a_ provided by the ASVCP^3^ or internal expert opinion (RETIC and PCT). QC validation, using the manufacturer's acceptable limits and observed analytical performance of analyzer 1, showed that only 3/10 measurands (HGB, WBC, and PCT) achieved an acceptable *P*_ed_ with the recommended TE_a_ or expert opinion in the case of PCT (Table 1). Consequently, the manufacturer's acceptable limits could not be relied on to identify clinically important equipment malfunction for most measurands. The wide manufacturer's limits were not unexpected, as this has been highlighted previously,^14^, ^15^ and these limits are generally derived from groups of instruments rather than individual instruments. For the manufacturer's limits that failed to achieve an acceptable *P*_ed_, error detection was as low as >0.01, using the closest available QC rule^6^, ^8^, ^15^ of 1‐5s or 1‐6s. In comparison, the QC validation used the observed performance of individual analyzers for all 10 measurands, achieving a *P*_ed_ >0.94 with the 1‐2.5s QC rule and a *P*_ed_ >0.85 with the 1‐3s QC rule for the observed performance of analyzer 1, resulting in much higher error detection.

We determined that a higher *P*_ed_ could be achieved using QC rules validated for each individual analyzer rather than the use of the manufacturer's acceptable limits. We determined that we could achieve an acceptable *P*_ed_, >0.94 for 95% of observations and *P*_fr_ ≤0.05 for all observations for our network of Sysmex analyzers. We failed to meet the *P*_ed_ criteria (>0.85, *n* = 1) for 3/60 (5%) of observations (analyzer 4a for RBC, PLT, and PCT) but were able to achieve the required *P*_ed_ >0.85 using either 1‐2.5s and/or 1‐3s QC rule, *n* = 1, for all other measurands. It is noteworthy that the performance of analyzer 4a improved following instrument service and was able to achieve the quality goals (data not shown). This is compared with the manufacturers' *P*_ed_, where only two measurands achieved >0.94 (Table 1). We are satisfied that the QC validation from the observed analytical performance offers the most consistent probability of error detection ≥0.85 for the largest number of measurands and, therefore, greater confidence in quality control and the likelihood of network stability. Using the manufacturer's acceptable limits does not provide sufficient *P*_ed_ for most measurands to have peace of mind regarding detection of unstable instrument performance. The probability of false rejection was within *P*_fr_ criteria (<0.05) for analyzer 1 using both manufacturer's limits and observed analytical performance. Overall, the individual analyzer validation allowed us the ability to achieve an error detection ≥0.85 and low levels of false rejection, resulting in more satisfactory QC using the 1‐2.5s rule.

The 1‐2.5s rule proved to be the optimal solution for one level of control material as the *P*_ed_ was >0.94 for the majority of observations, whereas a *P*_ed_ of 0.88 was the highest value achieved using the 1‐3s rule at the recommended TE_a_. This was achieved with little variation in *P*_fr_ between the rules used to assess individual analyzer validation since *P*_fr_ is considered a function of the rule used and the number of QCM and is not based on analyzer performance.

For the measurand (PCT) that failed to meet acceptable criteria for *P*_ed_ using both QC rules (1‐2.5s and 1‐3s), it was considered whether the TE_a_ based on expert opinion was too stringent; however, as the remaining analyzers performed optimally, declining performance was more likely. Moreover, on closer evaluation, the large CV (5.31%) and sigma metric <6 (σ = 4.56) suggest that for this measurand, the instrument performance was deteriorating and required attention, which was reflected in both QC rule failures.

Sigma metrics were applied as an additional performance monitoring tool and in conjunction with QC validation. The use of sigma metrics as a performance indicator is well documented.^16^, ^17^, ^18^ Sigma metrics were >6 for all measurands for analyzer 1 when based on observed analytical performance. For measurands on other analyzers that performed with <6 sigma (bias and/or imprecision for RBC and PLT on analyzer 4a was distinct from the other measurands across the instruments that performed at >6 sigma).

On closer evaluation for these measurands, we could determine limits for observed imprecision when applying a 1‐2.5s rule to achieve acceptable *P*_ed_. In reviewing the literature, we could not conclusively draw relevant comparisons due to the limited research in veterinary publications regarding imprecision goals. However, we believe that the following information is of interest to others performing QC validation using hematology analyzers. Biologic variation data were not available for PCT; however, if bias was set to 0%, then an observed CV ≤4.30% would be required to achieve an acceptable *P*_ed_ and >6 sigma; the observed CV for analyzer 4a was 5.31%. RETIC for analyzer 4b had a sigma metric of 5.98 but could achieve an acceptable *P*_ed_ and *P*_fr_ for the 1‐2.5s and 1‐3s rules; however, unlike the other measurands, the observed CVs were not distinct from the other analyzers. Biologic variation data were not available for this measurand, but from our data, if bias was 0%, an observed CV ≤7.0% is required to meet the quality goal (40%).

These observations demonstrate that, depending on the error budget, a low % CV with a high bias may still achieve the quality goal and vice versa, but they must meet desirable goals based on biologic variation. The use of sigma metrics in conjunction with the QC rules (Westgard Sigma rules) highlighted analytical instability for analyzer 4a compared with the other 5 analyzers and technical attention was required for those measurands performing at <5.5 sigma.

The measurands that were easily controlled were >5.5 sigma. These measurands had results with desirable biases and CVs based on biologic variation and could achieve high *P*_ed_ and low *P*_fr_ at the specified TE_a_. By meeting these criteria, these measurands were controlled using a simple QC rule. This is more cost‐effective^16^, ^18^ and less labor‐intensive for the technician, offering a degree of confidence to the clinical pathologist interpreting the results in a multisite, multi‐analyzer environment. For these measurands, a 1‐2.5s rule was considered the best candidate rule for ongoing use. This validation study is a much‐needed step in a harmonization process, ensuring that our network of analyzers is comparable. We know that analytical variability exists between instruments of the same model^7^, ^19^; therefore, by implementing validated QC rules and monitoring analytical performance using Westgards Sigma Rules,^20^ we are controlling this aspect of variability. The benefits gained from harmonization using maximally efficient high *P*_ed_ and low *P*_fr_ control approaches include a reduction of network costs, uniformity of standard operating procedures, unified quality management policy, unified training and proficiency, less rerun waste, and increased uniformity of turnaround times across laboratories.

Measurands more difficult to control were those <5.5 sigma, which had lower error detection (<0.85 *P*_ed_) and higher observed CV (>3.5%) compared with better performing measurands. In most cases, these measurands failed to meet the desirable CV based on biologic variation (Tables 2, 3, 4). In review of the criteria for identifying a need for analytical and technical attention, previous findings^7^ suggest that a bias >3%, failure to meet desirable biologic variation goals, and sigma metrics <5 were immediate triggers for poor performance requiring servicing. However, this study suggested some modifications to those criteria because some measurements showed suboptimal performance (PLT, RBC, and PCT for analyzer 4A) when sigma metrics were <5.5 rather than <5. For those example measurands with biological variation data available (RBC, HGB, HCT, WBC, and PLT), those that failed to meet desirable biologic variation goals with a sigma metric <5.5 warrant further investigation as they were poorly controlled with a 1‐2.5s and/or 1‐3s rule.

A more complicated multirule could be adopted in these cases, but in the interest of keeping QC simple and not too laborious for the technicians and network quality Managers, our recommended approach is to monitor the sigma metric, observed bias %, and observed CV %. We are confident that using (1) the 1‐2.5s QC rule, (2) ensuring that the desirable biologic variation‐based quality requirements for bias and CV are met, and (3) maintaining sigma metrics >5.5 sigma are excellent indicators of analytical stability sufficient to maintain harmonization. In addition, we continue to use nonstatistical measures such as a microscopic blood film evaluation by a hematologist, the consideration of clinical findings by a clinical pathologist, patient history, and a comparison/correlation of previous results. These measures are particularly important and recommended for measurands where statistical QC is not performed or does not provide high *P*_ed_. When statistical QC may not be sufficient to provide necessary quality monitoring, the addition of nonstatistical QC is recommended to help ensure that accurate and reliable results are reported.^6^

Some academic work has been published on QC validation of hematology analyzers, but this has predominantly been in human medicine.^21^, ^22^ In veterinary medicine, biochemistry analyzer QC validation has encompassed more of the focus.^14^, ^15^ Early work by Freeman and Gruenwaldt,^23^ as well as some comparative work,^24^, ^25^, ^26^ has created a good foundation for our harmonization study and emphasized that QC validation should be a requirement for veterinary laboratories, which is further supported in this study.

## 5 CONCLUSIONS

We recommend QC validation of individual hematology analyzer performance to achieve high *P*_ed_ and low *P*_fr_ rather than the use of the manufacturer's acceptable QC limits for hematology in veterinary laboratories. We validated 57/60 observations using the 1‐2.5s and/or 1‐3s QC rule; however, for optimal *P*_ed_, we applied the 1‐2.5s QC rule. This simple rule used with a single QCM is adequate for individual analyzers and for a harmonized network of analyzers if the sigma metrics remain >5.5 and the measurands meet desirable biologic variation goals for CV and bias. These standards should be applied across groups of analyzers and should aid the internal quality TE_a_m, pathologists, and technicians in recognizing when analytical issues arise.

## DISCLOSURE

The authors have indicated that they have no affiliations or financial involvement with any organization or entity with a financial interest in, or in financial competition with, the subject matter or materials discussed in this article.
